# Rituximab maintenance therapy for patients with diffuse large B-cell lymphoma: A meta-analysis

**DOI:** 10.1371/journal.pone.0174648

**Published:** 2017-03-29

**Authors:** Xuan Zhou, Tingting Ma, Yichan Zhang, Na Zhou, Juan Li

**Affiliations:** Department of Hematology, The Affiliated Drum Tower Hospital of Nanjing University Medical School, Gulou District, Nanjing, China; University of North Carolina at Chapel Hill, UNITED STATES

## Abstract

**Purpose:**

The addition of rituximab to standard chemotherapy has significantly improved survival in patients with lymphoma. Recently, maintenance therapy with rituximab has been shown to prevent relapse and provide survival benefits for patients with follicular or mantle cell lymphoma. However, the effects of rituximab in patients with diffuse large B-cell lymphoma (DLBCL) remain unclear. Two new studies involving rituximab in the treatment of DLBCL were performed this past year. We performed a meta analysis to evaluate the effects of rituximab maintenance treatment of patients with DLBCL.

**Methods:**

Several databases (PubMed, MEDLINE, EMBASE, and Cochrane Central Register of Controlled Trials) databases were reviewed for relevant randomized controlled trials published prior to May, 2016. Two reviewers assessed the quality of the included studies and extracted data independently. The hazard ratios (HRs) for time-to-event data and relative risks (RRs) for the other data were pooled and estimated.

**Results:**

Totally 5 studies including 1740 patients were eligible for the meta-analysis. Compared to the observation group, patients who received rituximab maintenance therapy had significantly improved event-free survival (EFS) (HR = 0.80, 95% CI: 0.65–0.98) and progression-free survival (PFS) (HR = 0.72, 95% CI: 0.54–0.94). However, there was no statistically significant difference in overall survival (OS) (HR = 0.66, 95% CI: 0.27–1.29). A subgroup analysis suggested that male patients may benefit from rituximab maintenance therapy with a better EFS (HR = 0.53, 95% CI: 0.34–0.82-), while this advantage was not observed in female patients (HR = 0.99, 95% CI: 0.64–1.52).

**Conclusions:**

Rituximab maintenance may provide survival benefits beyond that afforded by first- and second-line chemotherapy alone, especially in male patients. However, maintenance rituximab treatment may cause more adverse events. It is recommended that both survival benefits and adverse events should be taken into consideration when making treatment decisions.

## Introduction

Diffuse large B-cell lymphoma (DLBCL) is the most common type of non-Hodgkin's lymphoma (NHL) and accounts for more than 30% of all NHL cases[1]. The addition of rituximab to chemotherapy regimens has greatly improved survival for DLBCL patients regardless of first- or second-line treatment[2, 3]. Recently, greater attention has focused on the use of rituximab as maintenance therapy after treatment-induced remission. Rituximab maintenance treatment has been shown to improve progression-free survival (PFS) in patients with follicular lymphoma[4, 5]. Ren et al[6] analyzed the use of rituximab as maintenance or salvage therapy in DLBCL patients. They concluded that there was no statistically significant improvement in overall survival (OS) and event-free survival (EFS) in DLBCL patients using maintenance therapy. As additional studies have been reported recently, we performed a meta-analysis to evaluate the effects of rituximab maintenance in patients with DLBCL.

## Methods

### Identification and study selection

Two independent reviewers performed the literature search. Relevant trials were identified by searching multiple databases, including PubMed, MEDLINE, EMBASE, the Cochrane controlled trials register, the Cochrane Library, and the Science Citation Index. Search terms included “randomized control trial”, “diffuse large B-cell lymphoma” or “DLBCL”, “rituximab maintenance”. Similar terms were cross-searched. All studies published prior to May 2016 were eligible. The abstracts of all potentially relevant publications were reviewed. Studies that met the pre-specified criteria were selected for the analysis.

### Inclusion and exclusion criteria

The meta-analysis included DLBCL patients with untreated, relapsed, and refractory DLBCL who had reached complete remission (CR), unconfirmed complete remission (CRu), or partial remission (PR) after induced chemotherapy. All chemotherapy regimens, methods of administration, and dosages were included. The study type was randomized controlled trial with rituximab maintenance in one arm and observation only in the other arm.

We excluded ongoing studies, nonrandomized studies, and studies with 10 or fewer patients per study arm. If the same author reported results that were obtained from the same patient population in more than one publication, then only the most recent or most complete report was included in the analysis.

### Quality assessment and data abstraction

Two reviewers independently performed quality assessment using a 6-point scoring system according to the Cochrane Handbook (available at http://handbook.cochrane.org). Data were independent abstracted by each reviewer. If there was disagreement regarding extracted data, a consensus was reached by a third investigator.

### Statistical analysis

The extracted information was analyzed using STATA software version 12.0. For time-to-event data, the log hazard ratios (HRs) and their variances were estimated using the methods proposed by Parmar et al[7], if not provided directly. Heterogeneity was checked by a Q-test. A P-value < 0.1 was defined as heterogeneous. Heterogeneity was quantified using the I^2^ metric (I^2^<25%, no heterogeneity; I^2^ = 25–50%, moderate heterogeneity; and I^2^>50%, large or extreme heterogeneity). A random-effect model (DerSimonian—Laird method) and fixed-effect model (Mantel—Haenszel method) were employed to generate the pooled results. Stratified analyses were performed to investigate causes for the heterogeneity across studies. The stability of the combined results was evaluated by sensitivity analysis. All statistical tests were two-sided.

## Results

### Description of included trials

**Fig 1:**
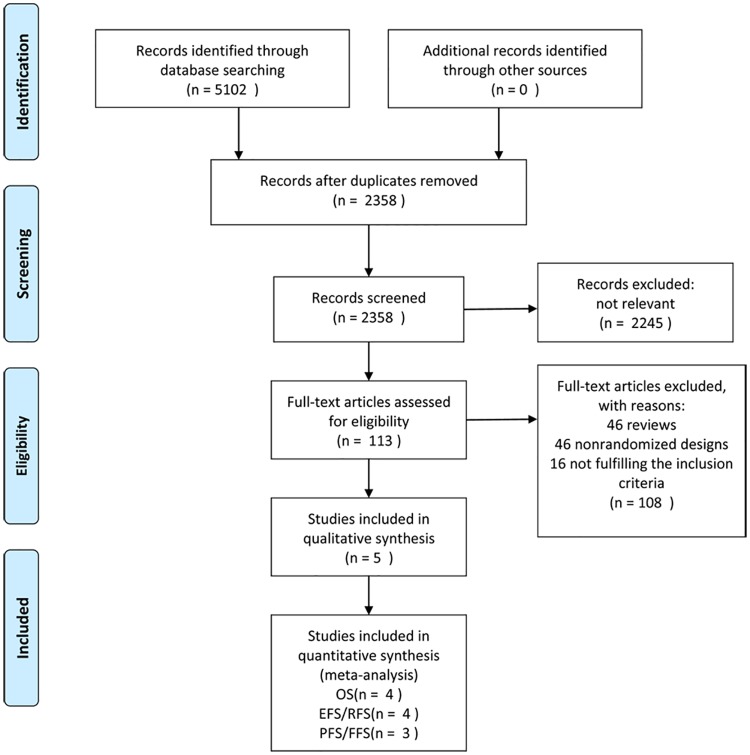
Flowchart of selection of studies for inclusion in meta-analysis.

A total of 113 potentially relevant publications were found using our search strategy (Fig 1). Among these, 39 were excluded for review, 53 were excluded for a nonrandomized design and 16 studies were excluded for not fulfilling the inclusion criteria. Overall, 5 RCTs fit the selection criteria and were included in this meta-analysis[8–12]. The baseline characteristics of the studies included are summarized in Table 1. Three trials focused on untreated DLBCL patients, one focused on relapsed or refractory patients, and the last one included both. ALL patients who were randomized to receive maintenance rituximab or observation alone achieved at least PR after induction therapy. EFS and RFS (relapse-free survival) were combined as the same outcome; PFS and FFS (failure-free survival) were also combined as an outcome. Four studies provided survival data for OS, four provided EFS (or RFS), and three provided PFS (or FFS). One trial[12] included 662 patients with DLBCL and 21 patients with follicular lymphoma grade 3b. According to a sub-analysis that was performed, “the results remained unchanged when only DLBCL patients were considered.” Consequently, we did not exclude the data of these 21 patients in our analysis.

**Table 1:** Characteristics of included studies.

references	No. of patients in meta-analysis (case/control)	Age (year)	histology	setting	Status at randomization	Prior therapy	maintenance	End-point	Median follow-up (month)
Habermann(2006)	415(207/208)	60–92	DLBCL	untreated	CR/PR	CHOP or R-CHOP	375 mg/m2/w X 4w every 6 mo for 2 y	FFS/ OS	40
Haioun(2009)	269 (139/130)	18–60	DLBCL/other high grade lymphoma	untreated	CR,/CRu /PR	ACVBP or AC/ACE 4 courses & ASCT	375 mg/m2/w X4w	EFS	48
Gisselbrecht(2012)	242(122/120)	18–65	DLBCL	Relapsed/Refractory	CR/CRu/PR (before ASCT)	R-ICE or R-DHAP & ASCT	375 mg/m2 every 8weeks for 1y	EFS/ OS	44
Jaeger(2015)	662(329/333)	>18	DLBCL/follicular lymphoma grade 3b	untreated	CR/CRu	R 8 courses & CHOP-like 4 to 8 courses	375mg/m2 every2 months for 6 doses or 12 doses (amendment)	EFS/ OS	45
Harig(2015)	152(77/75)	>18	DLBCL	Untreated/ Relapsed/Refractory	CR/PR	R-CHOP or other	375 mg/m2 every 3 months for 2 y	RFS/ OS	32

### Quality assessment

The quality of included studies was assessed according to the Cochrane Handbook (seen in Table 2). The standards of allocation concealment and blinding were nearly impossible to achieve in this analysis. Taking it into consideration, the scores ranging from 2 to 4 points were considered acceptable.

**Table 2:** Study quality.

Study	A	B	C	D	E	F	Total
Habermann(2006)	1	0	0	1	1	0	3
Haioun(2009)	1	0	0	1	1	0	3
Gisselbrecht(2012)	1	0	0	1	1	1	4
Jaeger(2015)	1	0	0	1	1	0	3
Harig(2015)	1	0	0	1	0	0	2

A: sequence generation; B: Allocation concealment; C: Blinding of participants, personnel and outcome assessors; D: Incomplete outcome data; E: Selective outcome reporting; F: Other potential threats to validity; 1: low risk; 0: high risk.

### Overall survival

**Fig 2:**
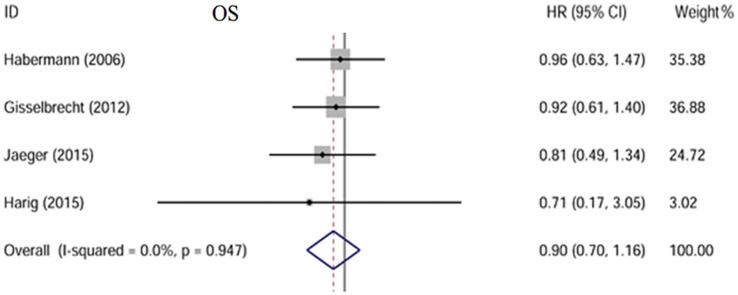
Forest plot of the HR. No significant difference of OS is observed between two groups; The size of the squares reflects each study’s relative weight, and the diamond (◊) represents the aggregate HR and 95% CI.

Four of the five trials included reported OS[8, 10–12] with a total of 735 patients in the rituximab maintenance arm and 686 in the observation arm. No statistical heterogeneity between studies was found (I^2^ = 0.0%, p = 0.947). We used a fixed-effect model. Patients in the maintenance arm did not have a significantly better OS than in the observation arm (HR = 0.66, 95% CI: 0.27–1.29) [Fig 2].

### Event-free survival

**Fig 3:**
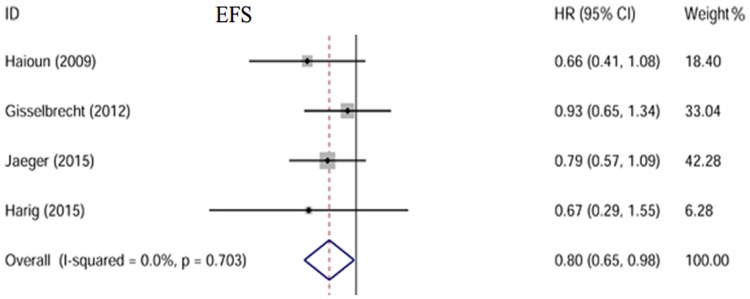
Forest plot of the HR. EFS is significantly improved in patients receiving rituximab maintenance.

Four trials including 1325 patients were analyzed with three reporting EFS[9, 10, 12] and one reporting RFS[11]. There was no obvious heterogeneity with I^2^ = 0.0% (p = 0.703). A fixed-effect model was used to perform the analysis. The results showed that EFS was improved in the maintenance arm (HR = 0.80, 95% CI: 0.65–0.98) compared to the observation arm [Fig 3].

### Progression-free survival

**Fig 4:**
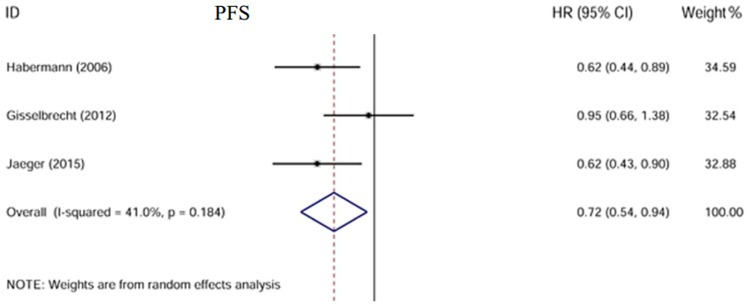
Forest plot of the HR. PFS is improved in patients in the maintenance arm.

Two studies reported PFS[10, 12] and one reported FFS[8]. A total of 1319 patients were included. There was moderate heterogeneity between these three trials with I^2^ = 41.0% (p = 0.184). A random-effect model was used. Our analysis showed PFS was significantly improved in patients who received rituximab maintenance treatment compared to those receiving observation only (HR = 0.72, 95% CI: 0.54–0.94)[Fig 4].

### Subgroup analysis

**Fig 5:**
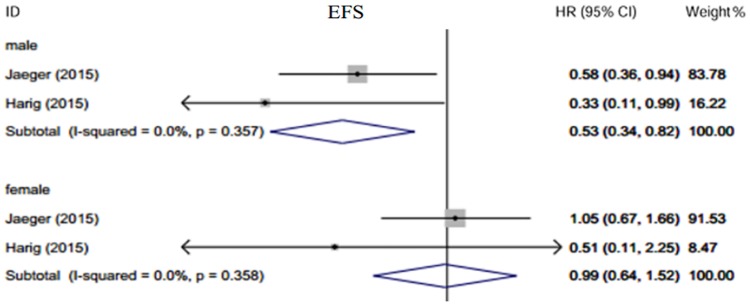
Subgroup analysis according to sex. Male patients benefit more in EFS than female from rituximab maintenance therapy.

Gisselbrecht et al.[10] reported that in the maintenance treatment group, women had greater EFS than men (70% vs 38%, p = 0.005). These gender-differential benefits were also observed when comparing OS and PFS in the maintenance arm (OS: 76% vs. 50%, p = 0.009; PFS: 70% vs. 48%, p = 0.005). However, the gender differences between the maintenance and observation groups were not analyzed. Jaeger et al.[12] compared the EFS and PFS of male patients between two arms. Male patients had better outcomes while female patients did not. Harig et al.[11] came to a similar conclusion and also found there was no gender difference in EFS in the maintenance group. We performed a subgroup analysis of the latter two trials using a random-effect model [Fig 5]. The results indicated greater survival benefits from rituximab maintenance in male patients (HR = 0.53, 95% CI: 0.34–0.82) compared to female patients (HR = 0.99, 95%CI: 0.64–1.52).

We did a subgroup analysis including only patients who had reached complete remission (CR) after induction chemotherapy. As shown in S1 Fig, patients who achieved CR had a tendency to have longer EFS (HR = 0.63, 95%CI: 0.33–1.18) in rituximab maintenance arm than in the observation arm. There were no obvious differences between the two treatment strategies for those who achieved CRu or PR (HR = 0.86, 95%CI: 0.51–1.44).--

### Adverse events

**Fig 6:**
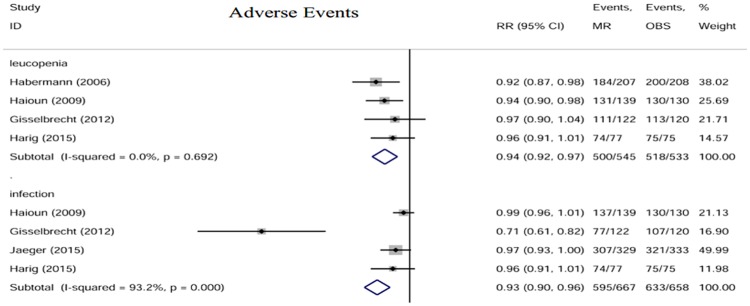
Forest plot of the RRs of main adverse events. The size of the squares reflects each study’s relative weight, and the diamond (◊) represents the aggregate RR and 95% CI. MR, rituximab maintenance; OBS, observation.

The main adverse events reported were Grade 3 or 4 leukopenia and infection. The data were pooled and analyzed [Fig 6]. There was a slight tendency for patients in the rituximab maintenance arm to have more adverse events than patients in the observation arm (leucopenia: RR = 0.94, 95% CI: 0.92–0.97; infection: RR = 0.93, 95% CI: 0.90–0.96).

### Sensitivity analysis

Our meta-analysis of PFS showed moderate heterogeneity between the trials. We conducted sensitivity analyses to check whether modification of the inclusion criteria of this meta-analysis affected the final results. A meta-analysis was performed after separately excluding every individual trial. We observed low heterogeneity when the study reported by Gisselbrecht[10] was excluded (I^2^ = 0.0%, p = 0.994), and the PFS improved substantially (HR = 0.62, 95% CI: 0.48–0.81)- [S2 Fig]. Since two trials[8, 12] included untreated patients and the other one[10] included relapsed or refractory patients, disease status may be an important factor that may affect patients’ outcomes. Consistent with this, we observe a difference in EFS between untreated patients (HR = 0.75, 95% CI: 0.57–0.98)- and those undergoing relapse (HR = 0.93, 95% CI: 0.65–1.34) [S3 Fig].- However, the OS remains similar (for untreated patients: HR = 0.90, 95% CI: 0.65–1.24;- for patients of relapse: HR = 0.92, 95% CI: 0.61–1.40) [S4 Fig].-

## Discussion

Rituximab is now used as the first- or second-line therapy in many kinds of lymphoma[13–15]. Its potential role in salvage and maintenance therapy has drawn a growing amount of attention[16, 17]. This meta-analysis combines the results from five trials compared rituximab maintenance with observation. Our pooled results suggest that maintenance rituximab therapy significantly improves PFS and EFS, but has no effect on OS. Subgroup analysis suggests that male patients, as well as untreated DLBCL patients, may benefit most from maintenance rituximab. However, there were relatively more adverse events reported in the rituximab group than the observation group.

Huang et al.[18] performed a retrospective analysis comparing rituximab maintenance to observation. Patients in the rituximab group had superior PFS. OS was also improved in patients with International Prognosis Index (IPI) ≥3. Another retrospective analysis was done by Zhong et al.[19] analyzing the efficacy of additional two-cycle rituximab for DLBCL patients in the first CR. It was interesting to know that additional rituximab prolonged PFS in patients with Revised-IPI low risk and National Comprehensive Cancer Network (NCCN)-IPI low risk. Our analysis suggests that rituximab maintenance may provide a certain degree of benefit to patients’ survival. However, adverse events increased due to long-term use of rituximab. This may explain why OS was not improved in the treatment arm. Moreover, the differences between EFS (or PFS) and OS are not solely based on toxicities, but also the ability to salvage patients after relapse. It remains possible that the heavy use of rituximab during the initial therapy for patients makes it more difficult to salvage them after relapse—contributing to the lack of an impact on OS compared with the control arm. The main adverse events were granulocytopenia and infection. It was recommended to delay the next cycle or discontinue the treatment if such an event occurred[20]. However, we did not find any publications referring to the management of these events. There was a review article[20] focused on the follow-up of DLBCL patients receiving rituximab. And no significant differences were seen in terms of treatment-related death between groups. Besides rituximab, many other compounds were under research. Peter et al.[21] investigated the use of maintenance thalidomide in patients with mantle cell lymphoma. Ongoing research is underway using different compounds as maintenance therapy for patients with DLBCL, such as immunomodulators and PD-1 inhibitors[22].

The gender differences in response to maintenance rituximab are particularly noteworthy[23]. Male patients typically have poorer prognosis with DLBCL than female patients[24]. Riihijarvi et al.[25] reported female patients had better PFS. However, Harig[11] and Jaeger[12] posed a challenge to this result. They argued that EFS was significantly improved in male patients but not in female patients. One possible explanation given was that female patients had a deeper remission during the pre-maintenance treatment. A new opinion was recently brought forward by Pfreundschuh et al.[26, 27]. They found that the speed of rituximab clearance in old female patients was reduced than in old male patients. This might partially explain why the effects of rituximab maintenance were not the same. Further studies are needed to investigate the relationship between the hemodynamic parameters of rituximab and outcomes of patients undergoing maintenance treatment. This may help identify subgroups of patients receive the maximum benefits from rituximab maintenance therapy.

Recently, quality of life (QoL) has been a focus for patients with lymphoma. One study[28] assessed QoL in patients with DLBCL prior to receiving R—CHOP treatment. This study reported a positive relationship between high QoL score and better outcomes. However, rituximab maintenance therapy has not been shown to significantly influence patients’ long-term QoL score[29]. QoL may serve as an independent index to evaluate the effect of rituximab maintenance treatment in future studies.

This analysis has several limitations. Firstly, heterogeneity is a potential problem when performing any meta-analysis. Heterogeneity can be caused by many factors, such as different inclusion criteria for the individual studies, different induction therapy, different rituximab administration protocols, and variable follow-up durations. It is difficult to perform subgroup analyses based on the factors mentioned above, which limits the value of the conclusions. Secondly, only published studies were included in this meta-analysis. Publication bias may exist. Lastly, we cannot give a convincing explanation on what we find through this meta-analysis because of few clinical trials. Whether rituximab has effects on patients of DLBCL still needs strict proofs.

In conclusion, rituximab maintenance after first-line treatment appears to benefit EFS and PFS, especially in male or previously untreated DLBCL patients. Our analysis suggests that these factors should be taken into account before a clinical decision is made. It is necessary for further studies to investigate whether special subgroups may particularly benefit from rituximab maintenance therapy and to elucidate the possible mechanisms.

## Supporting information

S1 Fig Subgroup analysis according to patients’ states (CR or CRu/PR) after induction therapy. A better status after induction therapy does not represent a better EFS.
(TIF)

S2 Fig Sensitivity analysis. No heterogeneity is observed after excluding Gisselbrecht’s study. And an improvement of PFS is observed.
(TIF)

S3 Fig Untreated patients benefit from rituximab maintenance with an apparently improved EFS. (TIF)

S4 Fig No improvement of OS is observed whatever disease status the patients are in. (TIF)

S1 Table PRISMA checklist 2009. (DOC)
