# Supplementary figures and images for: Rituximab maintenance therapy for patients with diffuse large B-cell lymphoma: A meta-analysis
Source: PLoS One. 2017 Mar 29;12(3):e0174648. doi: 10.1371/journal.pone.0174648 (PMC5371376; doi:10.1371/journal.pone.0174648)

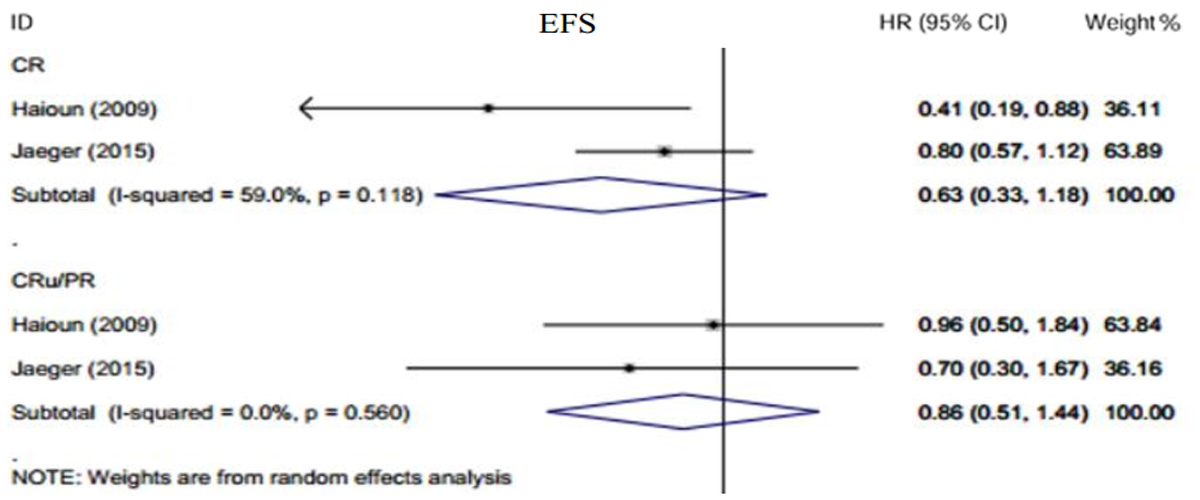

Supplement: S1 Fig — A better status after induction therapy does not represent a better EFS. (TIF) [file pone.0174648.s001.tif]

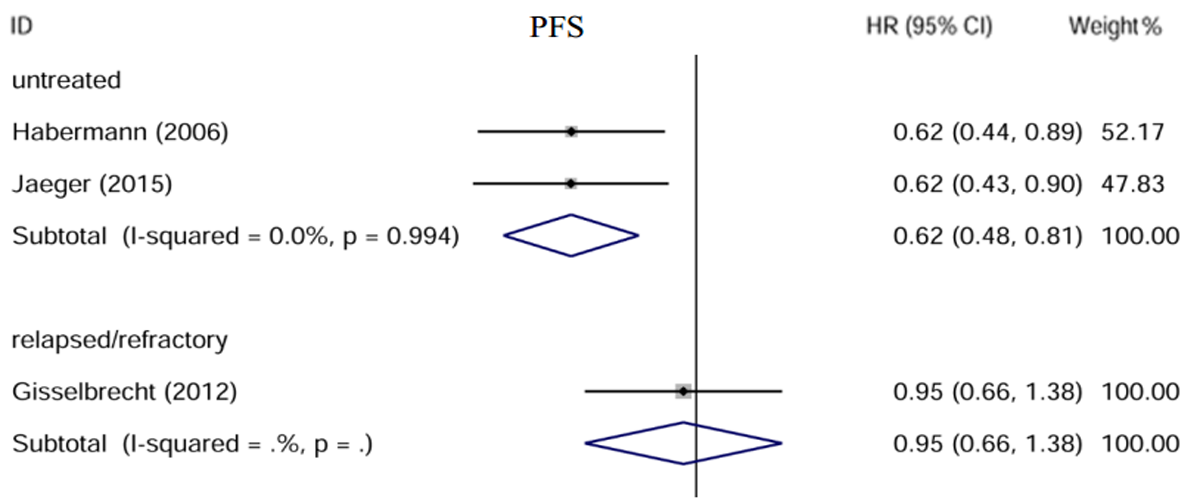

Supplement: S2 Fig — No heterogeneity is observed after excluding Gisselbrecht’s study. And an improvement of PFS is observed. (TIF) [file pone.0174648.s002.tif]

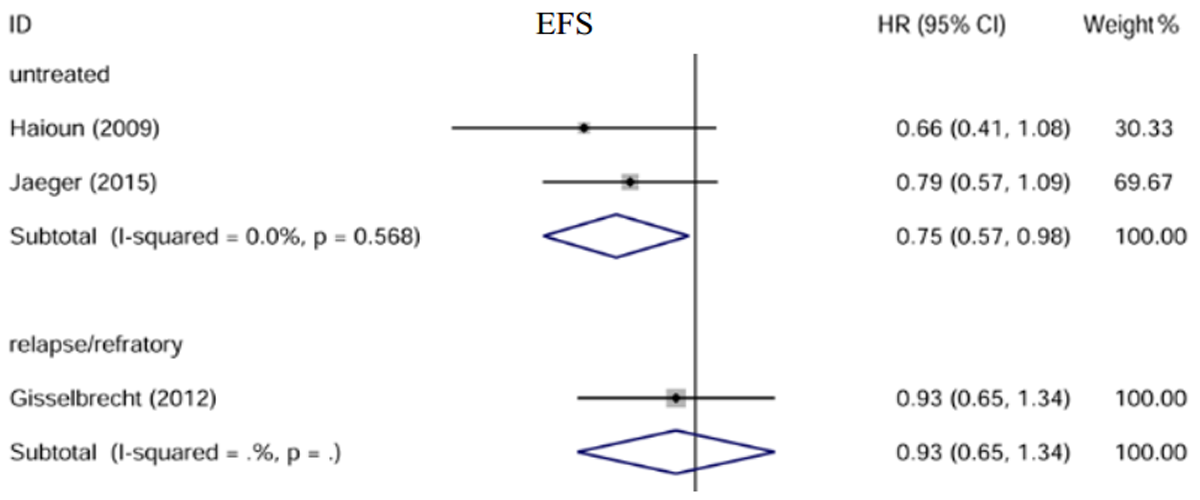

Supplement: S3 Fig — (TIF) [file pone.0174648.s003.tif]

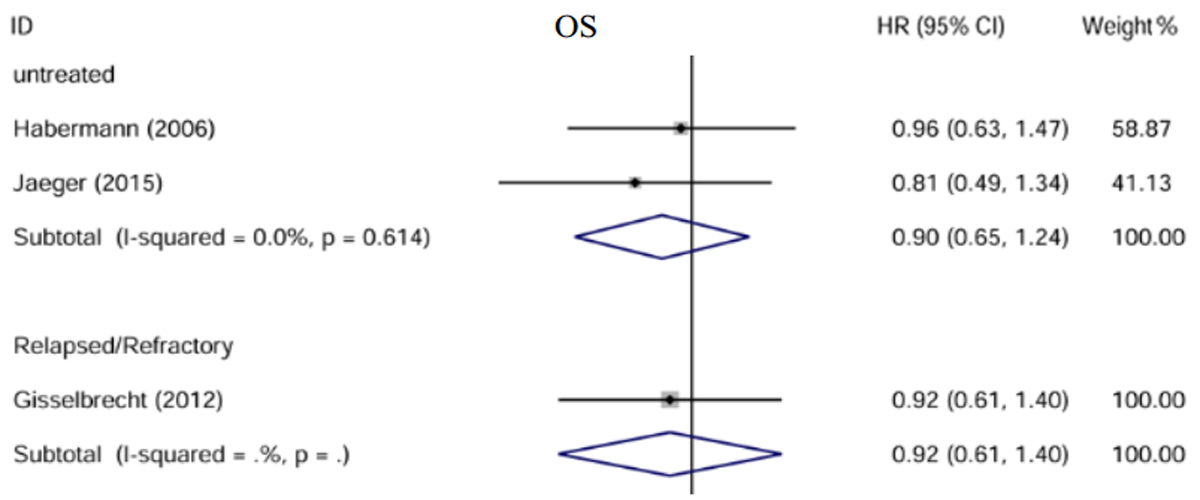

Supplement: S4 Fig — (TIF) [file pone.0174648.s004.tif]
